# Acute insular infarction: Early outcomes of minor stroke with proximal artery occlusion

**DOI:** 10.1371/journal.pone.0229836

**Published:** 2020-03-11

**Authors:** Seung-Hyun Min, Joon-Tae Kim, Kyung-Wook Kang, Min-Ji Choi, Hana Yoon, Yuki Shinohara, Michael H. Lev, Jeffrey L. Saver, Ki-Hyun Cho

**Affiliations:** 1 Department of Neurology, Chonnam National University Hospital, Gwanju, Korea; 2 Division of Radiology, Department of Pathophysiological and Therapeutic Science, Faculty of Medicine, Tottori University, Tottori, Japan; 3 Department of Radiology, Massachusetts General Hospital, Boston, MA, United States of America; 4 Department of Neurology and Comprehensive Stroke Center, David Geffen School of Medicine, University of California, Los Angeles, CA, United States of America; University of Ioannina School of Medicine, GREECE

## Abstract

**Background and purpose:**

We hypothesized that admission insular infarcts could be associated with early neurological deterioration (END) in acute minor stroke with large vessel occlusion.

**Methods:**

Using acute and follow-up diffusion-weighted imaging (DWI), we assessed insular involvement including the percent insular ribbon infarction (PIRI) scores and follow-up lesion patterns in acute minor stroke (NIHSS ≤5) with MCA/ICA occlusion. Follow-up lesion patterns were classified as swelling, new lesions, or infarct growth. END was defined as any increase in the NIHSS score.

**Results:**

Among 166 patients (age: 66±12 y, 60.8% male), 82 (49.4%) had insular lesions on baseline DWI, and 64 (38.6%) had PIRI scores ≥2. On follow-up DWI, infarct growths, new lesions, and swelling were observed in 34.9%, 69.9%, and 29.5% of patients. Infarct growths were significantly more frequent in patients with insular infarcts (43.9%), especially those with a PIRI score of 2 (54.8%), than in patients without insular infarcts (p = 0.02). While END was not significantly different in patients with and without insular lesions, insular lesions were independently associated with infarct growths (OR 2.18, 95% CI 1.12–4.26, p = 0.02) and END due to infarct growth (OR 2.54, 95% CI 1.12–5.76, p = 0.03), particularly in those with PIRI scores ≥2.

**Conclusion:**

In acute minor stroke with MCA/ICA occlusion, insular lesions on admission DWI, especially in patients with PIRI scores ≥2, were more likely to exhibit infarct growth and END due to infarct growth. This finding may help identify patients with higher risks of clinical worsening following acute minor stroke with large vessel occlusion.

## Introduction

Recent randomized studies demonstrated the efficacy of endovascular therapy (EVT) on the functional outcome of patients with acute ischemic stroke and large vessel occlusion.[1] However, the effects of EVT on functional outcome are still unknown in patients with low NIHSS scores (0–5) and large vessel occlusion. Therefore, acute treatment of patients with minor stroke should be determined based on specific features of individual patients, such as clinical or imaging findings.

Acute minor ischemic stroke with large artery occlusion results in relatively higher risks of early neurological deterioration (END) and poor outcomes.[2–4] Infarct growths and new infarcts in relevant arterial territory are considered the main mechanisms of END and poor outcomes in acute minor ischemic stroke with large artery occlusion. However, practical and reliable imaging biomarkers predictive of infarct growth or new infarcts in acute minor ischemic stroke with large artery occlusion have not been investigated. Accordingly, a rapid and intuitive prediction of the early radiological or clinical destination by initial imaging can aid therapeutic and preventive management.

Insular involvement in nearly half of patients with middle cerebral artery (MCA) territory infarcts has been associated with greater stroke severity, lesion growths with large mismatch losses, and poor outcomes of acute ischemic stroke.[5–8], The percent insular ribbon infarction (PIRI) score, which is a simple, practical visual assessment tool, has shown a significant predictive value for infarct growths and poor outcomes in acute MCA territory infarctions.[6, 9] However, data regarding the clinical implications of insular lesions on outcomes in acute minor stroke in MCA territory with large artery occlusion are sparse.

Therefore, we sought to investigate whether admission insular infarcts could be associated with early radiological and neurological outcomes in patients with acute minor stroke with large artery occlusion.

## Methods

This retrospective study is an analysis of registered patients with acute ischemic stroke in a single tertiary stroke center between March 2009 and December 2013. This study included patients who presented with acute minor infarcts of the MCA territory due to MCA/ICA occlusion within 6 hours of onset and were not treated with intra-arterial therapy. Minor infarction was defined as an NIHSS score of ≤5.[10] We excluded patients with (1) non-thrombotic etiologies of ischemic stroke, such as vasculitis and Moyamoya disease, and patients with cancer-related stroke; (2) prestroke disability, defined as a prestroke mRS score >1; and (3) an absence of or uninterpretable lesions on baseline/follow-up DWI. This study was approved by the institutional review board of Chonnam National University Hospital. Written informed consent was not obtained due to the retrospective nature of the study.

### Imaging assessment

**Fig 1:**
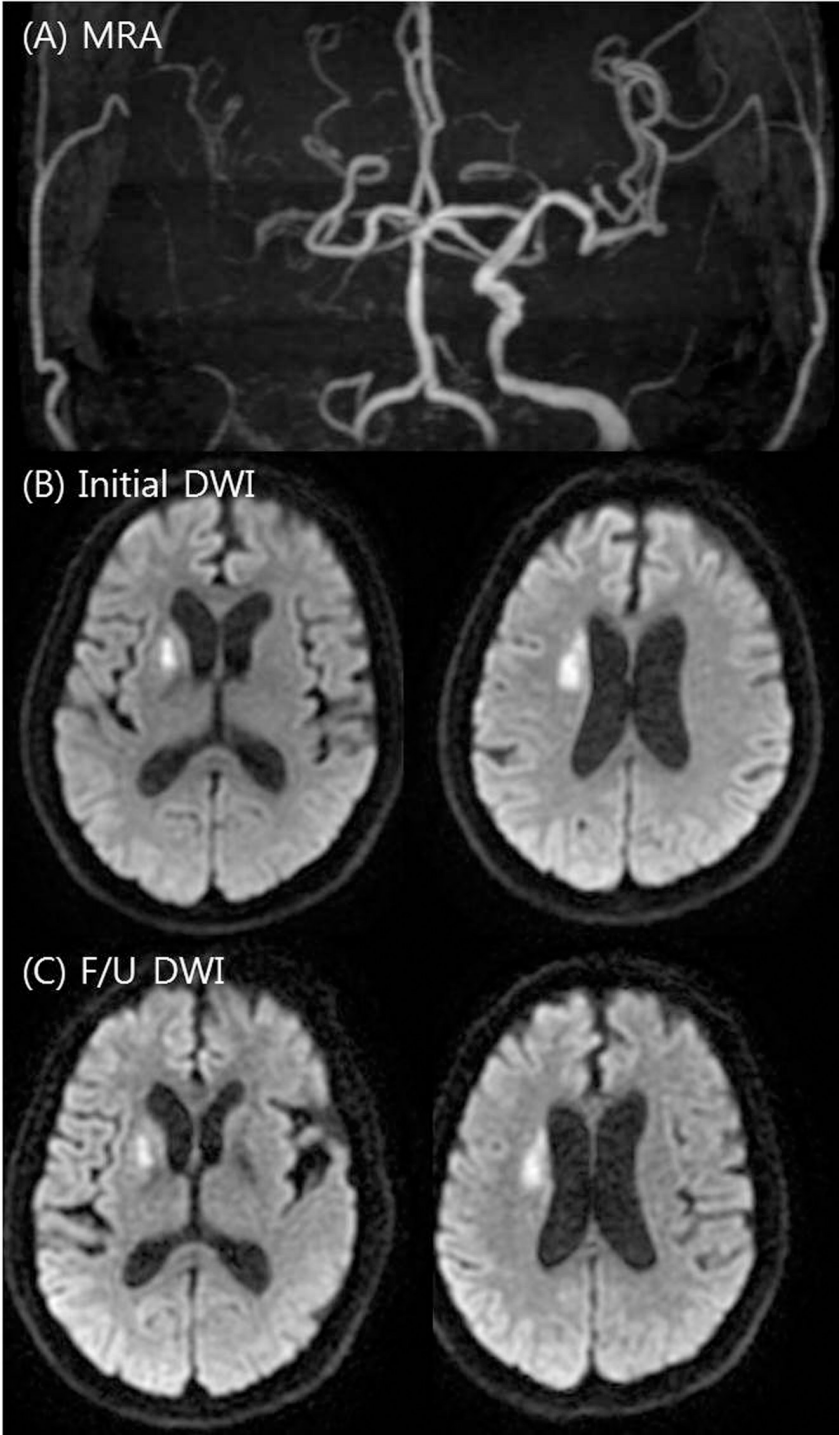
Example of a low percent insular ribbon infarction (PIRI) score. A 75-year-old man presenting 350 minutes after stroke onset with an NIHSS score of 1. MRI showed right internal carotid artery occlusion on time-of-flight intracranial MRA (A), with an ischemic lesion in the right putamen but a normal insula on initial DWI (B; PIRI score 0). On the follow-up DWI, no significant lesion changes were observed (C).

**Fig 2:**
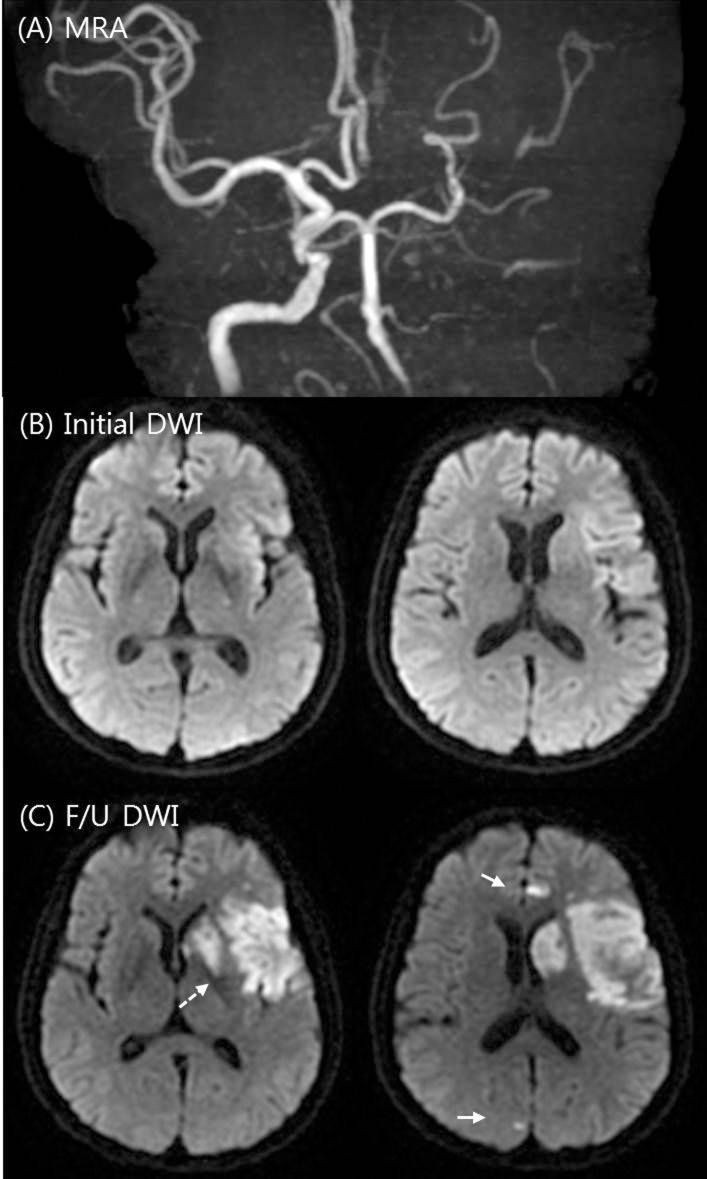
Example of a high percent insular ribbon infarction (PIRI) score. A 64-year-old man with an initial NIHSS score of 3 imaged 240 minutes after stroke onset. MRI showed left internal carotid artery occlusion on time-of-flight intracranial MRA (A), with subtle ischemic lesions with greater than 50% insular involvement on initial DWI (B; PIRI score 3). On the follow-up DWI (day 5), progressive insular infarction (dotted white arrow) with edematous changes and new ischemic lesions (white arrows) in the ACA and PCA territory were observed (C). Despite antiplatelet management, END occurred with an NIHSS score of 9.

The imaging protocol for acute ischemic stroke used in our hospital has been previously described. Briefly, patients underwent emergency MRI in the emergency department immediately after admission. The MRI protocol consisted of DWI, FLAIR, gradient echo imaging, time-of-flight MRA, and perfusion-weighted imaging in sequence. The MRI examinations were performed using a 1.5T unit (Signa HDxt; GE Healthcare, Milwaukee, Wisconsin). DWI sequences were obtained in the axial plane using a single-shot, spin-echo echoplanar technique with the following parameters: TR of 9000 ms, TE of 80 ms, section thicknesses of 4 mm, intersection gaps of 0 mm, FOVs of 260×260 mm, and b-values of 0 and 1000 s/mm^2^. Additionally, follow-up DWI imaging was routinely performed at day 5 and when END occurred. The patterns of baseline DWI lesions in the anterior circulation were classified as perforating artery infarcts (PAIs), pial infarcts (PIs), border zone infarcts (BIs), territorial infarcts (TIs), and lacunar infarcts (LIs) based on modifications to criteria of previous studies.[11] The PIRI score was independently rated on the admission DWI/ADC images according to a simple 5-point score that was based on percent involvement in quartiles (0, normal; 1, <25%; 2, 25%–49%; 3, 50%–74%; and 4, ≥75%).[6] Assessment was based on visual estimations using 3 contiguous axial slices depicting the longest extent of the insular ribbon. In addition, infarcts that involved the anterior, posterior, or both anterior and posterior insula were recorded. Follow-up DWI lesion changes were defined as 3 patterns with modifications from previous studies:[12, 13] infarct growth, new lesion, and lesion swelling. Infarct growth was defined as extension of the ischemic lesion in the same territory as that previously affected on the initial DWI, a new lesion was defined as the occurrence of a new lesion separate from initial lesions, regardless of the vascular territories of the initial lesions, and lesion swelling was defined as an edematous change of the initial lesion. Representative cases are shown in Figs 1 and 2. Multiple lesion patterns were separately rated for each pattern. For this study, the images were analyzed by two neurologists (J.-T.K. and K.-W.K.), each with more than 5 years of experience, who were blinded to all correlative clinical and other imaging data except laterality. Discrepancies were resolved by consensus.

### Clinical assessment

Demographic and clinical data were prospectively collected by dedicated research nurses or physicians. The following stroke risk factors were identified: age, sex, hypertension, diabetes mellitus (DM), dyslipidemia, atrial fibrillation, current smoking, and a previous history of stroke or TIA. Baseline data collected from all the patients included the NIHSS score and the stroke subtype, which was stratified according to the Trial of Org 10172 in Acute Stroke Treatment (TOAST) classification after complete diagnostic profiling.[14, 15] The NIHSS scores were assessed at admission and on each day of hospitalization by well-trained, dedicated stroke nurses.

END was defined as any neurological deterioration (i.e., any increase in the NIHSS score or the development of new neurological symptoms) during admission. For the sensitivity analyses, END-2 was defined as a 2-point or greater increase in the NIHSS score. Additionally, according to the follow-up DWI lesion patterns, END and END-2 were separately analyzed as follows: END with infarct growth, END with new lesions, and END with swelling.

### Statistical analysis

The percentages, means (±standard deviations, SD) or medians (interquartile range, IQR) were reported depending on the variable characteristics. Categorical variables were analyzed using the χ^2^-test and Fisher’s exact test as appropriate. Continuous variables were analyzed using the independent samples t-test or the Mann–Whitney U-test as appropriate. Multivariable logistic regression analyses were performed to evaluate the associations between early radiological/clinical outcomes and insular lesions (or PIRI scores of 2 or higher). The potential confounding variables included in these analyses were age, male sex, and baseline NIHSS score. Odds ratios (ORs) and 95% confidence intervals (CIs) were calculated. All P values were 2-sided, and statistical significance was defined as a p value less than 0.05. Statistical analyses were performed using SPSS for Windows version 17 (SPSS Inc., Chicago, IL, USA).

## Results

Among 527 patients who presented with acute minor stroke in the MCA territory within 6 hours of onset during the study period, 189 patients had relevant arterial occlusion in the MCA or ICA. Among them, 23 patients were excluded from the analysis for the following reasons: non-thrombotic etiologies of ischemic stroke (n = 8), prestroke disability (n = 8), incomplete or uninterpretable imaging (n = 3), and no outcome data (n = 4). Ultimately, 166 patients (mean age: 66±12 years, 60.8% male) were analyzed.

Among the 166 patients, 82 (49.4%) had insular lesions during baseline DWI, and 64 (38.6%) had PIRI scores ≥2. The median baseline NIHSS score was 2 (IQR 1–4). Tables 1 and S1 show the general characteristics of patients with and without insular lesions. Patients with insular lesions had a lower baseline Alberta Stroke Program Early Computed Tomography Score (ASPECTS) than those without lesions. Cardioembolism (CE) and undetermined (UD) categories, as determined using the TOAST classifications, as well as territorial infarct patterns on baseline DWI and distal MCA occlusion were more frequently observed in patients with insular lesions than in those without lesions. In contrast, large artery atherosclerosis (LAA) based on TOAST classifications, other infarct patterns except TI patterns on baseline DWI, and ICA occlusion were more frequently observed in patients without insular lesions. Patients with insular lesions had non-significantly higher baseline NIHSS scores than those without lesions (p = 0.12).

**Table 1:** General patient characteristics.

	No insular lesion (N = 84)	Insular lesion (N = 82)	p
Age (mean, SD)	67.3±11.9	66.5±12.6	0.66
Male	49 (58.3)	52 (63.4)	0.53
NIHSS (med, IQR)	2.0 (1, 4)	3.0 (2, 4)	0.12
Time to visit (min)	174±103	174±92	0.86
Hypertension	50 (59.5)	41 (50.0)	0.28
Diabetes mellitus	23 (27.4)	23 (28.0)	>0.99
Atrial fibrillation	26 (31.0)	32 (39.0)	0.33
Smoking	19 (22.6)	24 (29.3)	0.38
Dyslipidemia	15 (17.9)	22 (26.8)	0.19
Previous stroke or TIA	14 (16.7)	7 (8.5)	0.16
TOAST			**0.002**
LAA	48 (57.1)	25 (30.5)	
CE	27 (32.1)	38 (46.3)	
UD	9 (10.7)	19 (23.2)	
Initial DWI patterns			
PAI	17 (20.2)	4 (4.9)	**0.004**
PI	46 (54.8)	29 (35.4)	**0.013**
BI	26 (31.0)	10 (12.2)	**0.004**
TI	11 (13.1)	53 (64.6)	**<0.001**
LI	9 (10.7)	0	**0.003**
Occluded artery			**<0.001**
Distal MCA	24 (28.6)	49 (59.8)	
Proximal MCA	25 (29.8)	18 (22.0)	
ICA	35 (41.7)	15 (18.3)	
DWI-ASPECTS	9.0 (9, 10)	8.0 (7, 9)	**<0.001**

Abbreviations: TIA, transient ischemic attack; TOAST, Trial of Org 10172 in Acute Stroke Treatment; LAA, large artery atherosclerosis; CE, cardioembolism; UD, undetermined; DWI, diffusion-weighted imaging; PAI, perforating artery infarcts; PI, pial infarcts; BI, border zone infarcts; TI, territorial infarcts; LI, lacunar infarcts; MCA, middle cerebral artery; ICA, internal carotid artery; ASPECTS, Alberta Stroke Program Early CT Score.

Qualifying changes in lesions on follow-up DWI were observed in 145 (87.3%) patients. In the follow-up DWI, infarct growths were observed in 58 (34.9%) patients, new lesions were observed in 98 (69.9%) patients, and swelling was observed in 49 (29.5%) patients. Combined patterns of infarct growths and new lesions were observed in 20 (12.0%) patients, and swelling and new lesions were observed in 40 (24.1%) patients. Patients with insular lesions had higher frequencies of infarct growths on follow-up DWI than patients without insular lesions (43.9% vs. 26.2%, p = 0.02). However, new lesions and swollen lesions on follow-up DWI were not significantly different between patients with and without insular lesions. Additionally, infarct growths were most frequently observed for patients with PIRI scores of 2 (54.8%) than in those with other PIRI scores (Table 2). S2 Table shows the radiological and clinical outcomes according to the insular lesion locations (anterior/posterior/both anterior and posterior insula). No significantly different outcomes were observed for different insular lesion locations.

**Table 2:** Rates of early radiological and clinical outcomes according to the insular lesion and PIRI scores.

	No insular lesion (N = 84)	Insular lesion (N = 82)	p	PIRI = 0 (N = 84)	PIRI = 1 (N = 18)	PIRI = 2 (N = 42)	PIRI = 3–4 (N = 22)	p
Follow-up DWI patterns								
Infarct growth	22 (26.2)	36 (43.9)	**0.02**	22 (26.2)	5 (27.8)	23 (54.8)	8 (36.4)	**0.02**
New lesions	52 (61.9)	46 (56.1)	0.53	52 (61.9)	12 (66.7)	25 (59.5)	9 (40.9)	0.29
Swelling	20 (23.8)	29 (35.4)	0.13	20 (23.8)	5 (27.8)	15 (35.7)	9 (40.9)	0.32
END	32 (38.1)	32 (39.0)	>0.99	32 (38.1)	8 (44.4)	16 (38.1)	8 (36.4)	0.96
with infarct growth	10 (11.9)	21 (25.6)	**0.03**	10 (11.9)	4 (22.2)	13 (31.0)	4 (18.2)	0.08
with new lesions	23 (27.4)	16 (19.5)	0.27	23 (27.4)	6 (33.3)	7 (16.7)	3 (13.6)	0.27
with swelling	8 (9.5)	6 (7.3)	0.78	8 (9.5)	1 (5.6)	3 (7.1)	2 (9.1)	0.94
END-2	31 (36.9)	31 (37.8)	>0.99	31 (36.9)	7 (38.9)	16 (38.1)	8 (36.4)	0.99
with infarct growth	10 (11.9)	20 (24.4)	**0.04**	10 (11.9)	3 (16.7)	13 (31.0)	4 (18.2)	0.08
with new lesions	22 (26.2)	15 (18.3)	0.27	22 (26.2)	5 (27.8)	7 (16.7)	3 (13.6)	0.43
with swelling	7 (8.3)	6 (7.3)	>0.99	7 (8.3)	1 (5.6)	3 (7.1)	2 (9.1)	0.97

END, early neurological deterioration

END occurred in 64 (38.6%) patients, and END-2 occurred in 62 (37.3%) patients (Table 2). The numerical rates of END and END-2 were not significantly different between patients with and without insular lesions. However, END with infarct growth was more frequently observed in patients with insular lesions than in those without lesions (25.6% vs. 11.9%, p = 0.03). END with infarct growth was most frequently (but not significantly) observed for patients with a PIRI score of 2 among all PIRI scores. Excellent outcomes and good outcomes at 3 months were not different between patients with and without insular lesions, but patients with high PIRI scores (3–4) had the lowest frequency of good and excellent outcomes at 3 months (S3 Table).

For follow-up DWI lesion changes and early outcomes, insular lesions, compared with no insular lesions, were independently associated with infarct growth (OR 2.18, 95% CI 1.12–4.26, p = 0.02), END with infarct growth (OR 2.75, 95% CI 1.18–6.38, p = 0.02), and END-2 with infarct growth (OR 2.52, 95% CI 1.08–5.87, p = 0.03). Additionally, PIRI scores ≥2, compared with PIRI scores of 0–1, were independently associated with infarct growth (OR 2.57, 95% CI 1.30–5.07, p = 0.006), END with infarct growth (OR 2.54, 95% CI 1.12–5.76, p = 0.03), and END-2 with infarct growth (OR 2.72, 95% CI 1.19–6.24, p = 0.02) (Table 3). Regarding functional outcomes, insular lesions were non-significantly associated with reduced odds for a good outcome at 3 months (OR 0.63, 95% CI 0.32–1.25, p = 0.19) (S4 Table).

**Table 3:** Association between the presence of insular lesions (or PIRI score 2–4) and early radiological and clinical outcomes.

	Insular lesions (vs. no insular lesions)				PIRI 2–4 (vs. PIRI 0–1)			
	Crude OR* (95% CI)	p	Adjusted OR* (95% CI)	p	Crude OR* (95% CI)	p	Adjusted OR* (95% CI)	p
Follow-up DWI patterns								
Infarct growth	2.18 (1.12–4.26)	**0.02**	2.18 (1.12–4.26)	**0.02**	2.61 (1.35–5.04)	**0.004**	2.57 (1.30–5.07)	**0.006**
New lesions	0.76 (0.40–1.42)	0.39	0.76 (0.40–1.42)	0.39	0.67 (0.36–1.27)	0.22	0.63 (0.33–1.22)	0.17
Swelling	1.85 (0.93–3.69)	0.08	1.85 (0.93–3.69)	0.08	1.85 (0.94–3.64)	0.08	2.05 (1.01–4.15)	0.05
END	1.07 (0.56–2.03)	0.84	1.07 (0.56–2.03)	0.84	0.93 (0.49–1.77)	0.83	0.98 (0.51–1.92)	0.96
with infarct growth	2.75 (1.18–6.38)	**0.02**	2.75 (1.18–6.38)	**0.02**	2.27 (1.03–5.02)	**0.04**	2.54 (1.12–5.76)	**0.03**
with new lesions	0.63 (0.30–1.31)	0.21	0.63 (0.30–1.31)	0.21	0.47 (0.21–1.04)	0.06	0.44 (0.20–1.01)	0.05
with swelling	0.78 (0.25–2.42)	0.66	0.78 (0.25–2.42)	0.66	0.88 (0.28–2.74)	0.82	0.98 (0.30–3.20)	0.98
END-2	1.06 (0.56–2.01)	0.86	1.06 (0.56–2.01)	0.86	1.01 (0.53–1.93)	0.98	1.06 (0.54–2.06)	0.87
with infarct growth	2.52 (1.08–5.87)	**0.03**	2.52 (1.08–5.87)	**0.03**	2.48 (1.11–5.53)	**0.03**	2.72 (1.19–6.24)	**0.02**
with new lesions	0.61 (0.29–1.29)	0.19	0.61 (0.29–1.29)	0.19	0.51 (0.23–1.15)	0.11	0.48 (0.21–1.10)	0.08
with swelling	0.90 (0.28–2.87)	0.85	0.90 (0.28–2.87)	0.85	1.00 (0.31–3.19)	0.99	1.11 (0.33–3.71)	0.86

Adjusted variables; age, male sex, and baseline NIHSS.

*OR for each outcome in patients with insular lesions (ref. without insular lesions).

## Discussion

Our study found that patients with the presence of insular lesions, especially insular involvement over 25% (PIRI scores ≥2), were more likely to have infarct growths on follow-up DWI following acute minor stroke of MCA territories with MCA/ICA occlusion. In a separate analysis of END and 3 follow-up imaging changes, insular lesions were independently associated with a 2.5–2.8-fold increased risk of END/END-2 with infarct growths but were not associated with new infarcts and swelling. These results suggest that the presence of insular lesions in the baseline DWI, especially insular involvement over 25%, could be predictive of subsequent infarct growth and related END in patients with acute minor stroke with MCA/ICA occlusion.

Our study provides information that is helpful for understanding minor insular infarctions with MCA/ICA occlusion. The characteristics of insular infarcts shown in our study were consistent with those of previous studies, in which patients with insular involvement seemed to have more severe and larger strokes with higher baseline NIHSS scores, lower ASPECTSs, more frequent MCA occlusion, especially in M2, than ICA occlusion, and embolic or cryptogenic causes.[5, 7] These findings are reasonable based on the anatomical characteristics of the insular cortex as reported by previous studies.[5, 16] The arterial supply to insular regions is exclusively obtained from branches of the MCA, predominantly the M2 segment, and not from the pial collateral circulation of the anterior or posterior cerebral arteries.[16, 17] The anterior insula is supplied by superior M2 MCA division, whereas the inferior M2 division supplies the posterior insula.[16] Accordingly, the insular ribbon has high ischemic vulnerability to hypoperfusion.[18]

This study substantially expands the evidence indicating insular lesions as predictors of subsequent infarct growth in acute minor stroke. Here, we showed an independent association between insular lesions and infarct growths in acute minor stroke with large artery occlusion. Our results are consistent with those of a previous study in which insular lesions were more likely to progress into surrounding penumbral tissue in acute non-minor MCA infarcts.[7] Higher rates of infarct growths in insular infarcts than in non-insular infarcts could emphasize the importance of correlating radiological predictors with early neurological outcomes and subsequent follow-up outcomes.

Recently, neuroimaging parameters rather than clinical scores have been considered good predictors for subsequent outcomes, such as recurrent cerebrovascular events in minor strokes or TIAs.[19] Likewise, in acute minor stroke, infarct growths due to persistent large artery occlusion were key determinants of END and poor outcomes.[3] Accordingly, infarct growths rather than other patterns of change on follow-up DWI appeared to have more important prognostic implications for acute minor stroke. However, patients with cortical signs seemed more likely to progress to later infarct growth than those without these signs. Additionally, perfusion deficits are important predictors of infarct growth. However, our study focuses on the importance of the insular lesions for predicting infarct growth and END. Therefore, our results do not imply that imaging criteria alone are sufficient for initiation of mechanical thrombectomy in patients with insular lesions.

Our study provides supportive findings that insular involvement greater than 25%, defined as a PIRI score ≥2, results in a more than 2.5-fold higher risk of infarct growths in follow-up DWI than no or minor insular involvement (PIRI scores of 0–1). Notably, infarct growths were most frequently observed in more than half of patients with PIRI scores of 2. Because the PIRI scores were well correlated with the percent mismatch loss (infarct growths) in non-minor MCA infarction,[6] PIRI scores ≥2 could be considered important imaging markers of infarct growth in insular infarction with MCA/ICA occlusion, even in patients with low NIHSS scores. Furthermore, in patients with a small (≤70 mL) DWI infarct volume, a DWI-percentage insular ribbon infarct of greater than 50% (3–4) independently predicted a poor clinical outcome.[9] Because few patients had PIRI scores of 3–4, this group was underpowered in our study, and insular lesions, especially those in patients with higher PIRI scores, were associated with a trend toward a less favorable outcome at 3 months.

In addition, we analyzed the outcome of anterior insular infarct, posterior insula infarct, and infarcts that involve both the anterior and posterior portions of the insula. Although no significant different outcomes were observed for different insular locations, infarcts with both anterior and posterior insular lesions had numerically lower rates of good and excellent outcomes at 3 months. In a previous study,[5] temporary or permanent proximal M1 or ICA occlusion could result in the involvement of the lenticulostriate territory with major insular infarction or involvement of both the anterior and posterior portions of the insula. Also, as isolated anterior insula infarcts could be often worsened by other infarcts in the superior MCA division territory, whereas posterior insula infarcts also by inferior division infarction. Therefore, it is important to analyze the outcome of different insular locations; anterior, posterior or both anterior/posterior. However, further study is warranted to confirm these findings.

In the current study, 3 imaging patterns on follow-up DWI were used to estimate early radiological changes and probable mechanisms of END. Diverse mechanisms have been used to explain END, including collateral failure, clot progression, recurrent stroke, cerebral edema, hemorrhagic transformations and seizures, hemodynamic factors, excitotoxicity and inflammatory mechanisms.[20–24] However, because not all mechanisms could be shown to be relevant for END, we only considered follow-up DWI as a relevant determinant to objectively delineate the presumed mechanisms. A previous study reported that the clinical implications of unexplained END were determined by the initial penumbra and DWI lesion growths, i.e., progressive DWI lesions within and beyond the penumbra,[13] whereas our study considered follow-up infarct patterns, irrespective of the penumbral and extrapenumbral patterns, as infarct growth or new lesions based on the index lesions. Although the detailed mechanisms of END were different from those found in this study, the importance of infarct growth for END seems to be consistent with the results of a previous study. Further in-depth analyses of the possible mechanisms are warranted to confirm our results.

We found that insular lesions, compared with no insular lesions, were independently associated with 2-fold greater odds of END with infarct growth. These results suggest that potential treatment strategies could include protecting subsequent infarct growths to obtain good functional outcomes in patients with acute minor stroke with large artery occlusion. Rescue intra-arterial therapy could be a potential therapy to avert poor outcomes after END in patients with acute minor stroke with large artery occlusion.[25] However, our definition of END, i.e., any deterioration of the NIHSS score, could have been too sensitive to discriminate clinically relevant END. Nonetheless, in patients with acute minor stroke, a sensitive definition of END could be acceptable and lead to similar results as those obtained for END-2. While the rates of END were not different between patients with and without insular lesions, three-month good functional outcomes were non-significantly less frequent in patients with insular lesions, especially those with higher PIRI scores. Because ENDs were closely linked with clinical outcomes at 3 months, the clinical impacts of END in patients with insular lesions on functional outcomes at 3 months, especially for those with higher PIRI scores, may be more substantial than those in patients without insular lesions.

Our study has potential limitations. First, this study had an inherent limitation in that it was a retrospective, single-center study with a relatively small sample size. Second, residual or unmeasured confounding could be present; therefore, a statistical adjustment may be required. Third, the results of our study cannot be reasonably generalized for all patients with minor stroke because we only included patients with large artery occlusion in the anterior circulation stroke. Fourth, MRA-based determination of large vessel occlusion has inherent limitations including that the slow flow within the MCA or ICA may result the appearance of occlusion. In addition, we could not assess the rate of insula to cortex involvement, which might be another important imaging predictor for END. Limitations also existed because of not being quantified or volumetric analysis of perfusion imaging, only visual assessment of perfusion deficits. Therefore, the results of our study should be cautiously interpreted.

In conclusion, our study showed that patients with a presence of insular lesions exhibited more frequent infarct growth on follow-up DWI than those with an absence of insular lesions in acute minor anterior circulation infarction with large artery occlusion. Our results suggest that insular lesions, especially those with greater than 25% involvement, can be predictors for subsequent infarct growths and END. Subsequently, as such patients with acute minor stroke with large artery occlusion might be potential candidates for mechanical thrombectomy.

## Supporting information

S1 Table Characteristics of insular lesions. (DOCX)

S2 Table Rates of early radiological and clinical outcomes according to the insular lesion location. (DOCX)

S3 Table Rates of functional outcomes at discharge and 3 months according to the insular lesion and PIRI scores. (DOCX)

S4 Table Association between the presence of insular lesions (or PIRI score 2–4) and functional outcomes at discharge and 3 months. (DOCX)

S1 Data (XLSX)
