# Supplementary material for: Acute insular infarction: Early outcomes of minor stroke with proximal artery occlusion
Source: PLoS One. 2020 Mar 11;15(3):e0229836. doi: 10.1371/journal.pone.0229836 (PMC7065779; doi:10.1371/journal.pone.0229836)
Supplement: S1 Table — (DOCX) [file pone.0229836.s001.docx]

Supplemental Table 1. Characteristics of insular lesions

|  | No insular lesion | Insular lesion | p |
| --- | --- | --- | --- |
| N | 84 | 82 |  |
| Insular location, n (%) |  |  | NA |
| Anterior insula | NA | 37 (45.1) |  |
| Posterior insula |  | 37 (45.1) |  |
| Both |  | 8 (9.8) |  |
| Occluded artery |  |  | <0.001 |
| M2 superior div. | 8 (9.5) | 17 (20.7) |  |
| M2 inferior div. | 11 (13.1) | 28 (34.1) |  |
| M2 bifurcation | 0 | 4 (4.9) |  |
| M1 | 30 (35.7) | 18 (22.0) |  |
| ICA | 35 (41.7) | 15 (18.3) |  |
| Perfusion mismatch | 52/73 (71.2) | 49/71 (69.0) | 0.96 |
