# Supplementary material for: Acute insular infarction: Early outcomes of minor stroke with proximal artery occlusion
Source: PLoS One. 2020 Mar 11;15(3):e0229836. doi: 10.1371/journal.pone.0229836 (PMC7065779; doi:10.1371/journal.pone.0229836)
Supplement: S2 Table — (DOCX) [file pone.0229836.s002.docx]

Supplemental Table 2. Rates of early radiological and clinical outcomes according to the insular lesion location.

|  | No insular lesion | Anterior | Posterior | Both | p |
| --- | --- | --- | --- | --- | --- |
| N | 84 | 37 | 37 | 8 |  |
| FU DWI patterns |  |  |  |  |  |
| Infarct growth | 22 (26.2) | 17 (45.9) | 16 (43.2) | 3 (37.5) | 0.11 |
| New lesions | 52 (61.9) | 19 (51.4) | 23 (62.2) | 4 (50.0) | 0.66 |
| Swelling | 20 (23.8) | 11 (29.7) | 15 (40.5) | 3 (37.5) | 0.29 |
| END | 32 (38.1) | 15 (40.5) | 15 (40.5) | 2 (25.0) | 0.86 |
| with infarct growth | 10 (11.9) | 11 (29.7) | 8 (21.6) | 2 (25.0) | 0.12 |
| with new lesions | 23 (27.4) | 8 (21.6) | 7 (18.9) | 1 (12.5) | 0.63 |
| with swelling | 8 (9.5) | 1 (2.7) | 5 (13.5) | 0 | 0.30 |
| END-2 | 31 (36.9) | 15 (40.5) | 14 (37.8) | 2 (25.0) | 0.88 |
| with infarct growth | 10 (11.9) | 11 (29.7) | 7 (18.9) | 2 (25.0) | 0.12 |
| with new lesions | 22 (26.2) | 8 (21.6) | 6 (16.2) | 1 (12.5) | 0.58 |
| with swelling | 7 (8.3) | 1 (2.7) | 5 (13.5) | 0 | 0.29 |
| mRS 0-1 at discharge | 24 (28.6) | 7 (18.9) | 9 (24.3) | 1 (12.5) | 0.58 |
| mRS 0-2 at discharge | 49 (58.3) | 21 (56.8) | 19 (51.4) | 3 (37.5) | 0.66 |
| mRS 0-1 at 3 months | 45 (53.6) | 17 (45.9) | 17 (45.9) | 2 (25.0) | 0.42 |
| mRS 0-2 at 3 months | 62 (73.8) | 25 (67.6) | 23 (62.2) | 4 (50.0) | 0.38 |

END, early neurological deterioration; mRS, modified Rankin Scale.
