# Supplementary material for: Acute insular infarction: Early outcomes of minor stroke with proximal artery occlusion
Source: PLoS One. 2020 Mar 11;15(3):e0229836. doi: 10.1371/journal.pone.0229836 (PMC7065779; doi:10.1371/journal.pone.0229836)
Supplement: S3 Table — (DOCX) [file pone.0229836.s003.docx]

Supplemental Table 3. Rates of functional outcomes at discharge and 3 months according to the insular lesion and PIRI scores

|  | No insular lesion  (N=84) | Insular lesion  (N=82) | p | PIRI=0  (N=84) | PIRI=1  (N=18) | PIRI=2  (N=42) | PIRI=3-4  (N=22) | p |
| --- | --- | --- | --- | --- | --- | --- | --- | --- |
| mRS 0-1 at discharge | 24 (28.6) | 17 (20.7) | 0.28 | 24 (28.6) | 5 (27.8) | 9 (21.4) | 3 (13.6) | 0.48 |
| mRS 0-2 at discharge | 49 (58.3) | 43 (52.4) | 0.53 | 49 (58.3) | 11 (61.1) | 23 (54.8) | 9 (40.9) | 0.49 |
| mRS 0-1 at 3 months | 45 (53.6) | 36 (43.9) | 0.22 | 45 (53.6) | 10 (55.6) | 18 (42.9) | 8 (36.4) | 0.38 |
| mRS 0-2 at 3 months | 62 (73.8) | 52 (63.4) | 0.18 | 62 (73.8) | 12 (66.7) | 28 (66.7) | 12 (54.5) | 0.36 |

mRS, modified Rankin Scale.
