# Supplementary material for: Acute insular infarction: Early outcomes of minor stroke with proximal artery occlusion
Source: PLoS One. 2020 Mar 11;15(3):e0229836. doi: 10.1371/journal.pone.0229836 (PMC7065779; doi:10.1371/journal.pone.0229836)
Supplement: S4 Table — (DOCX) [file pone.0229836.s004.docx]

Supplemental Table 4. Association between the presence of insular lesions (or PIRI score 2-4) and functional outcomes at discharge and 3 months

|  | Insular lesions (vs. no insular lesion) | | | | PIRI 2-4 (vs. PIRI 0-1) | | | |
| --- | --- | --- | --- | --- | --- | --- | --- | --- |
|  | Crude OR*  (95% CI) | p | Adjusted OR*  (95% CI) | p | Crude OR*  (95% CI) | p | Adjusted OR*  (95% CI) | p |
| mRS 0-1 at discharge | 0.79 (0.36-1.72) | 0.55 | 0.79 (0.36-1.72) | 0.55 | 0.58 (0.27-1.24) | 0.16 | 0.70 (0.31-1.61) | 0.40 |
| mRS 0-1 at 3 months | 0.72 (0.37-1.38) | 0.32 | 0.72 (0.37-1.38) | 0.32 | 0.59 (0.31-1.10) | 0.10 | 0.61 (0.31-1.21) | 0.16 |
| mRS 0-2 at discharge | 0.79 (0.43-1.45) | 0.45 | 0.85 (0.44-1.65) | 0.62 | 0.70 (0.37-1.31) | 0.27 | 0.75 (0.38-1.49) | 0.41 |
| mRS 0-2 at 3 months | 0.62 (0.32-1.19) | 0.15 | 0.63 (0.32-1.25) | 0.19 | 0.63 (0.32-1.23) | 0.18 | 0.65 (0.33-1.31) | 0.23 |

Adjusted variables: age, male sex, and baseline NIHSS.

*OR for each outcome in patients with insular lesions (ref. patients without insular lesions).
